# Internal RNA Replication Elements are Prevalent in *Tombusviridae*

**DOI:** 10.3389/fmicb.2012.00279

**Published:** 2012-08-06

**Authors:** Beth L. Nicholson, Pui Kei K. Lee, K. A. White

**Affiliations:** Department of Biology, York University,Toronto, ON, Canada

**Keywords:** *Tombusvirus*, *Carmovirus*, *Necrovirus*, *Aureusvirus*, plant virus, RNA virus, RNA structure, RNA replication

## Abstract

Internal replication elements (IREs) are RNA structures that are present at internal positions in the genomes of different types of plus-strand RNA viruses. Members of the genus *Tombusvirus* (family *Tombusviridae*) contain an IRE within the polymerase coding region of their genomes and this RNA element participates in both genome targeting to sites of replication and replicase complex assembly. Here we propose that other members of the virus family *Tombusviridae* also possess comparable IREs. Through sequence and structural analyses, candidate IREs in several genera of this family were identified, including aureusviruses, necroviruses, carmoviruses, and pelarspoviruses. The results from subsequent mutational analysis of selected proposed IREs were consistent with a critical role for these structures in viral genome accumulation during infections. Our study supports the existence of IREs in several genera in *Tombusviridae* and points to previously unappreciated similarities in genome replication strategies between members of this virus family.

Plus-strand RNA viruses contain RNA elements within their genomes that regulate a variety of viral processes, such as translation, replication, encapsidation, and subgenomic mRNA transcription (Rao, 2006; Liu et al., 2009; Simon and Gehrke, 2009; Jiwan and White, 2011; Nicholson and White, 2011; Pathak et al., 2011). RNA sequences and structures located at the termini of viral genomes and complementary antigenomes are generally involved in modulating genome replication. However, it is becoming increasingly evident that some RNA elements involved in controlling replication may also be located internally, even within coding regions (Liu et al., 2009; Pathak et al., 2011). Such RNA elements, referred to herein as internal replication elements (IREs), generally correspond to functional local RNA secondary structures and have been identified in a variety of viruses including *Poliovirus* (Paul et al., 2000), *Hepatitis C virus* (You et al., 2004), and *Flock house virus* (Lindenbach et al., 2002), as well as plant bromoviruses (French and Ahlquist, 1987), dianthoviruses (Tatsuta et al., 2005), and tombusviruses (Monkewich et al., 2005).

Tombusviruses represent a well-developed model system for understanding *cis*- and *trans*-acting factors involved in plus-strand RNA virus genome replication (White and Nagy, 2004). There are two viral proteins necessary for tombusvirus genome replication; p33, an accessory replication protein, and p92, the RNA-dependent RNA polymerase (RdRp; Oster et al., 1998). The latter is a readthrough product of the former and the viral RdRp is present in the readthrough portion of p92 (**Figure 1A**). Tombusvirus RNA replication is initiated by association of p33/p92 with an IRE, termed RII(+)-SL (**Figure 1B**), located in the p92 coding region of the genome (**Figure 1A**; Pogany et al., 2005). The viral proteins target this ribonucleoprotein complex to peroxisomal membranes, the site of viral RNA synthesis (Panavas et al., 2005). Due to the 5′-proximal location of this IRE, it is not present within viral subgenomic mRNAs, thus genomes are selectively recruited for replication (Monkewich et al., 2005). Additionally, a long-range RNA–RNA interaction within the viral genome, involving complementary upstream and downstream linker sequences (UL and DL, respectively; **Figure 1A**), positions RII(+)-SL close to another important replication element in the 3′-terminus, termed RIV (**Figure 1A**). These united elements form a bipartite RII(+)-SL/RIV RNA platform that allows viral and host proteins to assemble into functional viral RNA replicase complexes (Panaviene et al., 2005; Wu et al., 2009).

**FIGURE 1:**
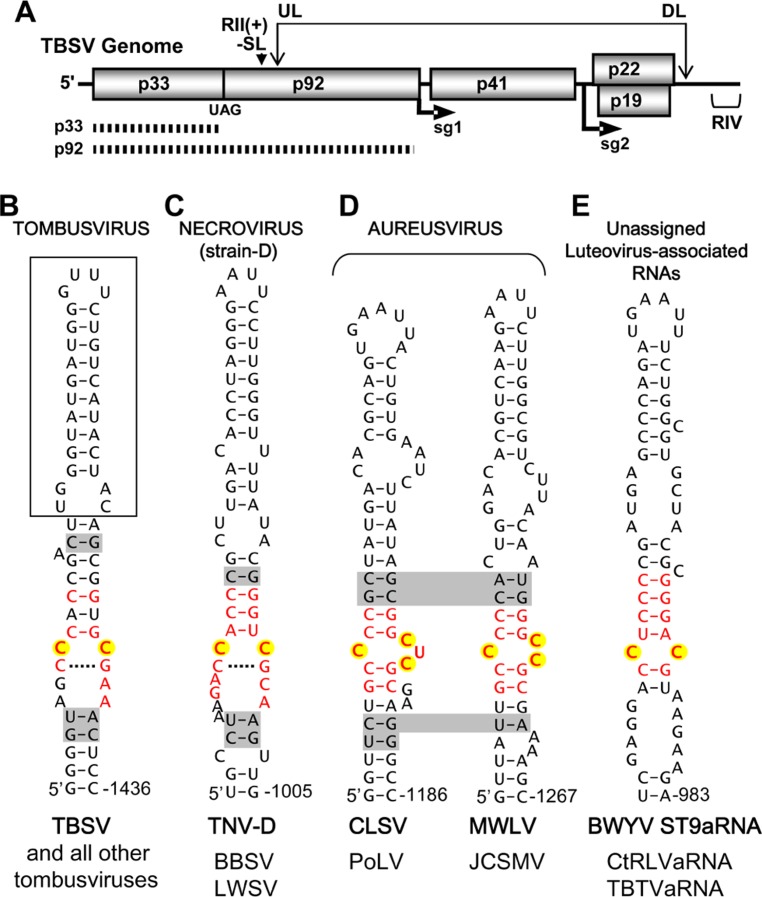
***Tomato bushy stunt virus* genome structure and proposed IREs in tombusvirus-like tombusvirids**. **(A)** Linear representation of the TBSV RNA genome showing coding regions and encoded proteins. The positions of initiation for subgenomic (sg) mRNAs 1 and 2 are shown under the genome. The replication proteins p33 (accessory replication protein) and p92 (RdRp, produced via readthrough) are depicted as hatched bars under the genome. The positions of important replication elements in the genome, the RII(+)-SL (i.e., the IRE) and RIV are indicated by a downward arrowhead and bracket, respectively. The long-range RNA–RNA interaction between complementary UL and DL sequences is indicated by the double-headed arrow. **(B)** *Tombusvirus* IRE. The established IRE for TBSV, termed RII(+)-SL is shown. The boxed region is dispensable for TBSV defective interfering RNA replication and a lower region of the structure not shown is essential for function (Monkewich et al., 2005). **(C)** Proposed strain D-like *Necrovirus* IRE. **(D)** Proposed *Aureusvirus* IREs. **(E)** Proposed *Luteovirus*-associated RNA IRE. In all cases, the IRE shown is from the virus indicated in bold and, below, genus members that share that particular structure are listed. The nucleotides in internal loop motifs that are conserved in each genus are shown in red, nucleotides corresponding to the CC mismatch in tombusviruses are highlighted in yellow, and base pairs shaded in gray represent positions of covariation within species of a genus that share the structure. Shading that traverses two structures indicates covariation at corresponding positions in the two structures shown. Tombusvirus: TBSV, *Tomato bushy stunt virus*. Necrovirus (strain D): TNV-D, *Tobacco necrosis virus*-strain D; BBSV, *Beet black scorch virus*; LWSV, *Leek white stripe virus*. Aureusvirus: CLSV, *Cucumber leaf spot virus*; PoLV, *Pothos latent virus*; MWLV, *Maize white line virus*; JCSMV, *Johnsongrass chlorotic stripe mosaic virus*. Luteovirus-associated RNAs: BWYV ST9aRNA, *Beet western yellows virus*-ST9 strain associated RNA; CtRLVaRNA, *Carrot red leaf virus*-associated RNA; TBTVaRNA, *Tobacco bushy top virus*-associated RNA.

*Tombusvirus* is the prototype genus of the virus family *Tombusviridae*, which includes seven additional official genera; *Aureusvirus*, *Avenavirus*, *Carmovirus*, *Dianthovirus*, *Necrovirus*, *Machlomovirus*, and *Panicovirus* (Sit and Lommel, 2010) and one provisional genus *Pelarspovirus* (Kinard and Jordan, 2002; Castaño et al, 2009). All, except for the dianthoviruses, use translational readthrough of the 5′-proximal accessory replication protein to produce their RdRps (**Figure 1**; Cimino et al., 2011; Tajima et al., 2011). Phylogenetic analysis of the RdRps in *Tombusviridae* indicates that tombusvirus RdRps are more closely related to those of aureusviruses and strain D-like necroviruses, whereas the carmovirus RdRps correlate more closely with those of panicoviruses, machlomoviruses, strain A-like necroviruses, and pelarspoviruses (Castaño and Hernández, 2005). Interestingly, the RdRp grouping of strain D-like necroviruses with tombusvirus-like viruses is somewhat unexpected, because necrovirus genomes resemble carmovirus genomes; i.e., they encode two small movement proteins and have the same gene organization (Coutts et al., 1991).

Although the RdRps of genera in *Tombusviridae* can be divided into subgroups, overall the RdRps of all members in this family exhibit a high degree of similarity (Castaño and Hernández, 2005). This suggests that *Tombusviridae* family members, termed tombusvirids (Vetten and Haenni, 2006), may also share some features related to genome replication. In terms of *cis*-acting RNA elements, non-segmented tombusvirid genomes possess a similar general 3′-terminal configuration, which involves a pseudoknot that embeds a common 3′-terminal CCC_-OH_ within a double-stranded region (Na and White, 2006). As described above, in tombusviruses, this pseudoknot-containing 3′-terminal region (i.e., RIV) functions in conjunction with an IRE [i.e., RII(+)-SL] to mediate replication (Panaviene et al., 2005; Wu et al., 2009). Based on this requirement in the type genus in this virus family, we wondered whether other tombusvirids also contained tombusvirus-like IREs. Accordingly, we investigated this possibility by carrying out RNA sequence/structure and mutational analyses on the members of this virus family with the goal of providing evidence for potential IREs.

The RII(+)-SL IRE in the tombusvirus type species *Tomato bushy stunt virus* (TBSV), which is also present in other members of this genus, forms an extended stem-loop RNA structure containing a CC mismatch that is a key determinant of p33 accessory replication protein binding and RNA genome replication (**Figure 1B**; Monkewich et al., 2005; Panaviene et al., 2005). We initiated our analysis by examining the genomic RNA sequences of tombusvirid genomes at regions that corresponded to the position of RII(+)-SL in tombusviruses. Based on RdRp relatedness, this region represented the most probable locale for identifying equivalent IREs. Mfold RNA secondary structure prediction analysis (Zuker, 2003) was performed on the sequenced genomes of viruses from each genus. Potentially relevant local RNA secondary structures were identified in necroviruses, aureusviruses, carmoviruses, and pelarspoviruses. Conversely, no compelling corresponding RNA structures were identified in avenaviruses, panicoviruses, or machlomoviruses. For this latter group, the limited number of species in each genus and the greater divergence of their RdRps contributed to the difficulty in confidently predicting IRE candidates. In the case of the segmented dianthoviruses, an IRE was previously identified in the movement protein coding region of genomic RNA2 in *Red clover necrotic mosaic virus* (Tatsuta et al., 2005), thus this genus already has a known IRE, albeit distinct from that in tombusviruses.

The criteria used to define new candidate IREs in the other tombusvirids included (i) mfold-predicted formation of the IRE in full-length genomes, (ii) base pair covariation within the IRE that maintained predicted RNA structures, and (iii) conservation of internal loop motifs similar to that in RII(+)-SL in tombusviruses. Additional support for candidate IREs in necroviruses and carmoviruses came from the observation that their locations correlated with previously reported RdRp coding regions that exhibited greater than expected synonymous site conservation (Firth et al., 2011). Such comparatively lower rates of substitution at degenerate codon positions imply conservation of an RNA secondary structure element within the coding segment (Firth et al., 2011). Based on the above criteria, IREs with the common general structure of an extended stem-loop were predicted for necroviruses, aureusviruses, carmoviruses, and pelarspoviruses. Additionally, a common IRE structure was identified for luteovirus-associated RNAs, which encode RdRps related to tombusviruses and are components of complex viral diseases that also include poleroviruses and umbraviruses (Passmore et al., 1993; Watson et al., 1998; Mo et al., 2011).

The proposed IRE for strain D-like necroviruses contained a central core structure that somewhat resembled that in tombusviruses, i.e., it contained an internal loop with a CC mismatch that was bounded by an upper stem and potential CG base pair below (**Figure 1C**). For aureusviruses, a central motif involving C residues was also evident; however, depending on the virus, one of two variations, involving either a C/CUC or C/CC internal loop, was present (**Figure 1D**). Thus, potential IREs in tombusvirids with tombusvirus-like RdRps contain a common motif corresponding to an internal loop with mismatched C residues. A similarly structured motif containing a CC mismatch was also identified in luteovirus-associated RNAs (**Figure 1E**).

For the large carmovirus genus, two distinct conserved motifs were identified within their corresponding extended stem-loop RNA structures. The first motif (M1) was U/AU containing a UA base pair and an unpaired U located in an upper region of the structure (**Figure 2A**). The second motif (M2), CAUXCC/GGZAGG (X = any nucleotide: Z = A, C, or U), was more extensive and formed an internal loop bounded by CG base pairs. M2 somewhat resembled the tombusvirus motif, i.e., the X and Z residues were bounded by an upper CG base pair and a potential lower UA base pair (versus CG in tombusviruses) and in most cases either X or Z was a C residue (while both corresponding positions in tombusviruses are C residues; compare **Figures 1B and 2A**). M2 was also present in strain A-type necroviruses, which have carmovirus-like RdRps (**Figure 2B**), and for both carmoviruses and strain A-type necroviruses, the predominant mismatch at the XZ positions was UC or AC (**Figures 2Ai,ii,B**). Additionally, members of the pelarspovirus genus, which also possess carmovirus-like RdRps, harbored a similar M2 with a UC at the XZ positions (**Figure 2C**). The XZ positions diverged from either UC or AC in only two carmoviruses. In *Angelonia flower break virus* (AnFBV), XZ was a CA mismatch, and in *Pelargonium flower break virus* (PFBV), XZ was a GU wobble pair (**Figures 2Aiii,iv**). Further differences in M2 were found only in *Cardamine chlorotic fleck virus* (CCFV), where the CG bounding the upper part of the internal loop was a UG wobble pair and the AG mismatch in the internal loop was a CG pair (**Figure 2Aii**). Overall, M1 was maintained in carmoviruses and strain A-type necroviruses but not in pelarspoviruses, while M2 was maintained in all three of these genera.

**FIGURE 2:**
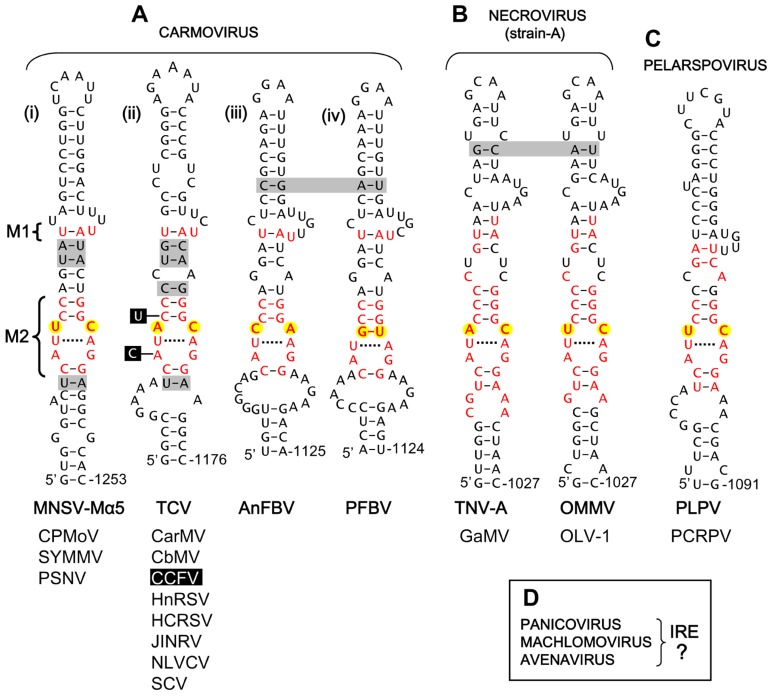
**Proposed IREs in carmovirus-like tombusvirids**. **(A)** Proposed *Carmovirus* IREs. **(B)** Proposed strain A-like *Necrovirus* IREs. **(C)** Proposed *Pelarspovirus* IRE. **(D)** Genera of *Tombusviridae* for which no putative IREs were identified. M1, motif 1, M2, motif 2. The residues in black boxes indicate substitutions present only in CCFV. See legend of **Figure 1** for more information. Carmoviruses: MNSV-Mα5, *Melon necrotic spot virus-Malpha5*; CPMoV, *Cowpea mottle virus*; SYMMV, *Soybean yellow mottle mosaic virus*; PSNV, *Pea stem necrosis virus*; TCV, *Turnip crinkle virus*; CarMV, *Carnation mottle virus*; CbMV, *Calibrachoa mottle virus*; CCFV, *Cardamine chlorotic fleck virus*; HnRSV, *Honeysuckle ring spot virus*; HCRSV, *Hibiscus chlorotic ring spot virus*; JINRV, *Japanese iris necrotic ring virus*; NLVCV, *Nootka lupine vein-*clearing virus; SCV, *Saguaro cactus virus*. AnFBV, *Angelonia flower break virus*; PFBV, *Pelargonium flower break virus*. Necrovirus (strain A): TNV-A, *Tobacco necrosis virus*-strain A; GaMV, *Galinsoga mosaic virus*; OMMV, *Olive mild mosaic virus*; OLV-1, *Olive latent virus-1*. Pelarspovirus: PLPV, *Pelargonium line pattern virus*; PCRPV, *Pelargonium chlorotic ring pattern virus*.

Having identified candidate IREs in several different genera of *Tombusviridae*, we next sought to acquire some supportive experimental evidence for their functional relevance. To this end, the strain D-type necrovirus *Tobacco necrosis virus-*D (TNV-D), the aureusvirus *Cucumber leaf spot virus* (CLSV), and the carmovirus *Turnip crinkle virus* (TCV) were selected for mutational analysis. Substitutions were introduced into the infectious clone of each of these viruses at degenerate codon positions within the proposed IREs. These substitutions did not cause alterations in the amino acid sequences of the encoded RdRps; with the exception of the A-to-C substitution in CLSV, which was a conservative glutamic acid to aspartic acid substitution (**Figure 3B**). The mutant genomes were then transfected into cucumber protoplasts and the relative levels of mutant genome accumulation, versus that for the wild-type virus, were determined (**Figure 3**). For TNV-D, which is more similar to tombusviruses, all substitutions within the conserved internal loop motif that modified the conserved CC mismatch completely inhibited virus accumulation (**Figure 3A**). In contrast, mutations outside the core motif were tolerated, indicating a critical role for the CC mismatch in TNV-D accumulation (**Figure 3A**). Similarly, changing one of the C residues in the core motif in CLSV to either A or G residues abolished genome accumulation, while a substitution at a more distal site was tolerated (**Figure 3B**).

**FIGURE 3:**
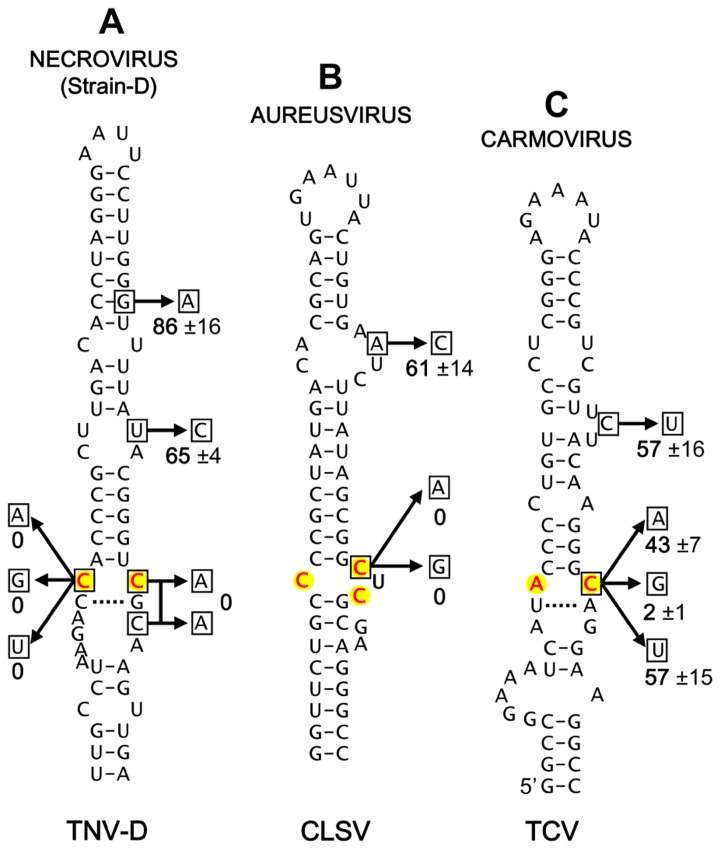
**Mutational analysis of selected proposed IREs**. **(A)** Mutational analysis of the proposed IRE in the *Necrovirus* TNV-D. **(B)** Mutational analysis of the proposed IRE in the *Aureusvirus* CLSV. **(C)** Mutational analysis of the proposed IRE in the *Carmovirus* TCV. Substitutions were introduced into the IREs of infectious clones of the respective viruses at degenerate codon positions. Wild-type and mutant viruses were transfected into cucumber protoplasts and following a 22-h incubation total RNA was harvested and separated in 1.4% agarose gels as described previously (Oster et al., 1998). Subsequent northern blot analysis was performed to detect the viral genomes, and their levels were quantified (Oster et al., 1998). The substitutions made in each structure are shown (boxed residues) and the relative accumulation level (±tandard error) for each mutant, calculated as a percentage of wild-type genome levels from three separate experiments, is indicated.

For TCV, changing the C residue in the AC mismatch to either A or U resulted in approximately 50% reductions in genome accumulation, whereas substitution with a G residue essentially eliminated genome accumulation (**Figure 3C**). The tolerance of some changes at this C position indicates a degree of flexibility in the motif, which is consistent with the observed naturally occurring A and U, but not G, substitutions at this position in other carmoviruses (**Figures 2Aiii,iv**). Therefore, unlike tombusviruses, aureusviruses and strain D-type necroviruses, all of which maintain a C at this 3′-position of the internal loop, carmoviruses appear to have diverged and are flexible with respect to this requirement (a concept further confirmed by verifying the stable maintenance of the C-to-A and C-to-U substitutions in progeny TCV genomes from infections via sequencing). This difference in carmoviruses suggests a greater functional role for other residues within M2 and/or M1. Moreover, the conservation of comparable motifs in strain A-type necroviruses and pelarspoviruses indicates similar functional requirements in these viruses. Collectively, the results of the mutational analysis are consistent with the proposed roles for the candidate IREs in genome replication and suggest that these viruses share aspects of their genome replication strategies with tombusviruses.

The results support our hypothesis that IREs are prevalent in *Tombusviridae* and they provide a foundation for further studies into the role of these newly identified IREs in viral genome replication. However, these findings do not preclude the possibility of the involvement of different or additional IREs located elsewhere in these genomes. Based on the known activities of the tombusvirus IRE, several questions related to the proposed IREs come to mind, including: Do these IREs bind specifically to their cognate pre-readthrough accessory replication proteins? Do these IREs communicate with replication elements at the 3′-terminus of their genomes? Are other regions of these IREs also important for their function? and Do these IREs mediate genome selection for replication or facilitate replicase assembly? Our results also prompt additional questions, such as: Are yet to be identified IREs present in panicoviruses and machlomoviruses? and Do comparable IREs extend to related umbraviruses or perhaps even luteoviruses? Future detailed structure/function analyses of the proposed *Tombusviridae* IREs will ultimately define the determinants of their function as well as their precise role(s) in virus reproduction.

## Conflict of Interest Statement

The authors declare that the research was conducted in the absence of any commercial or financial relationships that could be construed as a potential conflict of interest.
